# Improvement of Anorgasmia and Anejaculation After Spinal Manipulation in an Older Man With Lumbar Stenosis: A Case Report

**DOI:** 10.7759/cureus.34719

**Published:** 2023-02-07

**Authors:** Robert J Trager, Anthony Baumann

**Affiliations:** 1 Chiropractic, Connor Whole Health, University Hospitals Cleveland Medical Center, Cleveland, USA; 2 College of Chiropractic, Logan University, Chesterfield, USA; 3 Rehabilitation Services, University Hospitals Cleveland Medical Center, Cleveland, USA; 4 Medical School, Northeast Ohio Medical University, Rootstown, USA

**Keywords:** erectile dysfunction, ejaculation, orgasmic disorder, lumbar spine, spinal manipulation, chiropractic

## Abstract

A 72-year-old man with cardiovascular disease, depression, and anxiety presented to a chiropractor with a six-year history of anorgasmia, anejaculation, and erectile dysfunction as well as chronic, episodic low back pain. He previously saw a neurologist, two urologists, and had extensive and expensive testing, including brain, cervical, thoracic, lumbar, and pelvic imaging and electrodiagnostic testing. The patient had a disc bulge at L5/S1 causing moderate spinal canal stenosis while other testing was relatively normal. He had previously tried discontinuing a selective serotonin reuptake inhibitor, trialing psychological counseling, and administering penile injections, all without any improvement in sexual function. The chiropractor identified lower extremity weakness, sensory, reflex, and balance deficits and initiated a one-month trial of care, applying lumbar mobilizations and thrust manipulation at L1/2. The patient reported resolution of anorgasmia and anejaculation the first week, which was maintained over a total three months’ follow-up. Low back pain also did not return. The current case report highlights the apparent success of lumbar spinal manipulation in improving anorgasmia and anejaculation in an older man. This response may be explained in that the sympathetic (T10-L2) and somatic (S2-4) innervation required for male orgasm and ejaculation is derived from the lumbosacral region. Further research is needed to determine if these findings are reproducible.

## Introduction

Severe lumbosacral spine disorders, such as the emergency condition of cauda equina syndrome, may produce prominent neurologic deficits such as bladder dysfunction, perineal numbness, lower extremity neurological deficits and pain, as well as sexual dysfunction [1-2]. However, more common and less severe degenerative disorders of the lumbar spine, including disc displacement (i.e., herniation or bulging) or stenosis (i.e., narrowing), are also associated with sexual dysfunction [1-4]. This phenomenon has been suggested to result from the compression of autonomic or somatic nerves in the lumbosacral region which are required for sexual function [2-3].

In the literature, a case series of 38 males (median age: 66 years) undergoing lumbar spine surgery for stenosis found that 90% of these patients had erectile dysfunction [3]. Another surgical series of 22 males (mean age: 41 years) with acute lumbar disc herniation reported that 32% of patients had reduced ejaculation; however, the percentage of patients with this symptom reduced significantly after surgery (9%) [1]. A recent case series described four men with sexual dysfunction caused by lumbar disc bulging or herniation at L5/S1, all of whom had no low back pain [2]. Finally, a survey study including 25 men with acute L5/S1 disc herniation found that 92% of subjects reported reduced satisfaction with their sex life [4].

Unfortunately, male ejaculatory disorders are often disregarded or undiagnosed [5]. With respect to males with low back disorders, such patients typically ask their providers about physical activity and pain, and may avoid inquiring about sexual dysfunction [1]. In addition, spine care providers may have limited knowledge of the relationship between the lumbar spine and sexual dysfunction [1].

Spinal manipulation includes a range of therapeutic techniques directed to the joints of the spine, such as non-thrust mobilization, and manipulation with a thrust/impulse. These therapies are often used to treat musculoskeletal disorders of the spine such as low back pain [6]. However, to our knowledge, and via Google Scholar and PubMed searches as well as a recent review article [7], no studies have reported improvements in male anorgasmia or anejaculation with spinal manipulation.

Despite the known association between lumbosacral disorders and sexual dysfunction in men, there is limited research which describes the improvement of sexual dysfunction with conservative treatment of the lumbosacral spine. Therefore, we report a case of an older man wherein lumbar spinal manipulation appeared to improve anorgasmia and anejaculation.

## Case presentation

Patient information

A 72-year-old man with a history of hypertrophic cardiomyopathy, benign essential hypertension, glaucoma, depression, anxiety, and insomnia presented to a chiropractor in 2022 with a six-year history of anorgasmia, anejaculation, erectile dysfunction, and occasional episodes of mild to moderate local low back pain and stiffness. The patient was a non-smoker, occasional drinker, and worked full-time in finance. He noted that his low back pain was severe 20 years prior yet had improved significantly over the span of a few years without surgery or spinal intervention. At the time of the current visit, his low back pain was absent. However, the patient suspected his anorgasmia and anejaculation were related to his lower back dysfunction, considering he tried several other therapies unsuccessfully, but had not yet received treatment for his low back. He denied having any nocturnal emissions, genitourinary pain, bladder/bowel dysfunction, perineal or perianal numbness, or claudication symptoms with standing/walking. The patient’s Oswestry Disability Index was scored at 12% (minimal disability).

The patient was currently taking atorvastatin 40 mg, diltiazem 240 mg, losartan 100 mg, gabapentin 600 mg, levothyroxine 75 micrograms (mcg), aspirin 81 mg, a B-complex vitamin, coenzyme Q10 100 mg, fish oil 600 mg, and vitamin C 1000 mg. He previously took sertraline 200 mg (a selective serotonin reuptake inhibitor) for several years yet had tapered down and discontinued this medication under advice from his primary care provider that it may help with his anorgasmia. However, upon discontinuing this medication three months prior to his chiropractic appointment, he noticed no change in his anorgasmia or erectile dysfunction. The patient occasionally self-administered TriMix penile injections (i.e., alprostadil, papaverine, and phentolamine) to facilitate erections, yet reported this treatment did not enable him to have orgasms. He was advised by his cardiologist that he could not take sildenafil due to his cardiovascular disease and current medications (i.e., hypertension and diltiazem).

The patient’s anorgasmia, anejaculation, and erectile dysfunction began suddenly six years prior, after developing severe pneumonia and shingles. During this time, he developed a 48-hour episode of difficulty walking and was admitted to the hospital where he was treated with levofloxacin and valaciclovir. A neurologist then ordered brain CT, which was reportedly normal. After recovering from pneumonia and shingles, he had ongoing unsteadiness with walking, anorgasmia, and erectile dysfunction, which prompted him to see another neurologist. This provider noted hyporeflexia in the upper extremities and atrophy of the extensor digitorum brevis, and considered a broad differential diagnosis including lumbar stenosis, lumbosacral radiculopathy, lumbosacral plexopathy, pudendal neuropathy, lymphoma, pelvic abscess, thoracic cord lesion, multiple sclerosis, cerebral infarction, and cervical stenosis.

**Figure 1:**
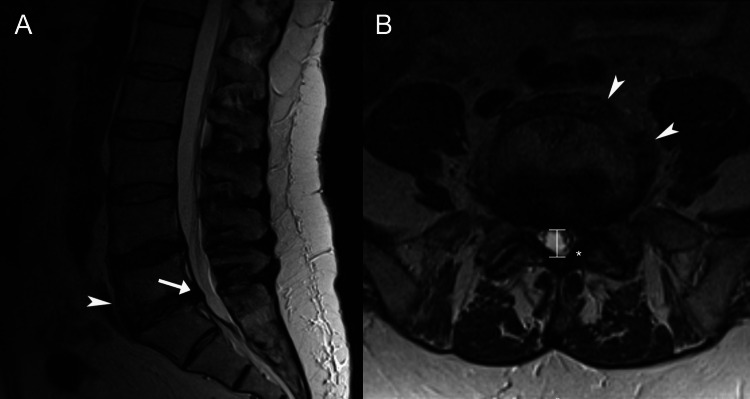
Lumbar MRI. The T2-weighted mid-sagittal view (A) demonstrates disc displacement at L5/S1 which measures 6 mm in anteroposterior diameter (arrow). The axial view of L5/S1 (B) better demonstrates a central disc bulge at this level, causing moderate spinal canal stenosis and moderate left and mild right neural foraminal narrowing. The anteroposterior diameter of the spinal canal (“I” marker) is narrowed to nine millimeters. In addition, the ligamentum flavum (*) is slightly thickened, measuring 5 mm in thickness on the left side, and contributes to the canal stenosis. As the axial view is not perfectly parallel with the disc plane, anterior osteophytes are also well visualized in this view (arrowheads), which measure a maximum 10 mm from the vertebral body. The disc bulge at L4/5 is smaller and does not cause any significant foraminal narrowing (not indicated).

**Figure 2:**
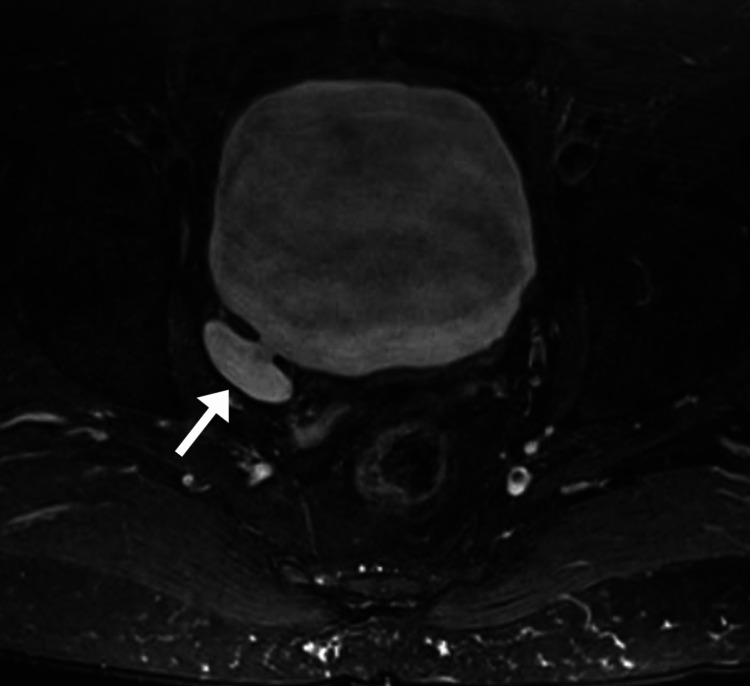
Axial lumbosacral plexus MRI. This T2 sequence oriented through the mid-pelvis shows the bladder measuring 9.4 cm x 8.9 cm with a small diverticulum (arrow) which was deemed to be incidental by the neurologist and urologist.

The neurologist ordered MRI of the brain, which revealed minimal nonspecific bi-hemispheric white matter signal changes. Lumbar spine MRI revealed a small disc bulge at L4/5 and diffuse posterior disc bulge at L5/S1 (Figure 1). A lumbosacral plexus MRI without and with contrast was normal, aside from a bladder diverticulum which was determined to be an incidental finding and did not require treatment (Figure 2). Cervical MRI revealed mild multifocal degenerative changes. A thoracic MRI was normal. The neurologist also ordered electromyography (EMG) and nerve conduction testing which showed chronic moderate left-sided L4 and L5 radiculopathies and chronic mild right-sided L4 and L5 radiculopathies, both without active denervation. No polyneuropathy was noted. The neurologist determined there was no significant neurological disease aside from a lumbar radicular disorder and referred the patient to a urologist.

The urologist ordered a prostate specific antigen test which was normal (0.42 ng/mL) and pelvic CT which again revealed the small urinary bladder diverticulum. The urologist suspected the patient’s anorgasmia was psychogenic and subsequently referred him to a psychologist. The patient sought the opinion of another urologist specializing in erectile dysfunction, who also suggested the patient’s symptoms were psychogenic. The patient then attended several counseling sessions over a span of multiple months and was reportedly informed by the psychologist that his anorgasmia was not psychogenic. The patient’s primary care provider also ordered several laboratory tests which were normal, including total testosterone (612 ng/dL), vitamin D (36 ng/mL), thyroid stimulating hormone (3.23 mIU/L), serum prolactin (9.4 μg/L), a hepatic function panel, and Lyme disease antibody screen.

Clinical findings

Upon examination by the chiropractor, six years after the patient’s pneumonia and shingles, the patient was observed to have moderately limited lumbar range of motion, obvious atrophy of the extensor digitorum brevis on both feet, and an increased thoracic kyphosis. There was diminished sensation to light touch in the second through fifth toes of both feet. The patient was unable to abduct his toes on either foot, and had 3/5 strength (Medical Research Council Scale) of toe extension bilaterally while great toe extension was normal. The triceps surae muscle stretch reflexes were 1+ bilaterally while the patellar and medial hamstring reflexes were normal (2+). There were no upper extremity sensory, motor, or reflex deficits and no pathological reflexes (i.e., Hoffman, Babinski). Motion palpation revealed tenderness and limited mobility at the spinal segments L1/2. During the Romberg test for balance with the eyes closed, the patient lasted three seconds before swaying forwards and needing to open his eyes to prevent himself from falling.

Due to the deficits in sensation, strength, and hyporeflexia in the lower extremities along with a balance deficit, as well as previously identified lumbar disc displacement and stenosis at L5/S1, the chiropractor considered a working diagnosis of lumbosacral radiculopathy. Therefore, the chiropractor recommended a trial of care consisting of lumbar spinal manipulative therapy, to which the patient consented.

**Figure 3:**
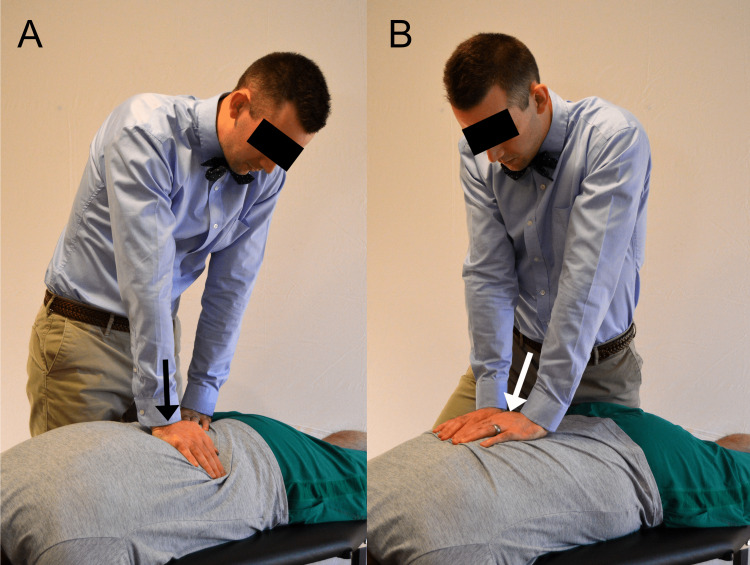
Demonstration of lumbar spinal mobilization and manipulation. In image A, the practitioner applies posterior-to-anterior mobilization. The practitioner’s left hand stabilizes the pelvis, while the right hand provides oscillatory movement of a large amplitude with a low velocity, moving into the area(s) of stiffness (black arrow). In image B, the practitioner provides a single, high-velocity, low-amplitude thrust with a posterior to anterior and slightly superior vector at the level of the first lumbar vertebra (white arrow).

At the first visit, the chiropractor applied non-thrust mobilizations to L5, L4, and L3, and the sacroiliac joints bilaterally for approximately three minutes, and a single high-velocity, low-amplitude thrust manipulation to L1/2 (Figure 3). The patient subjectively reported that later that week he was able to have an orgasm with ejaculation, achieved through self-stimulation on his own accord. We did not assess any objective measure or questionnaire documenting these changes. The patient returned for his second visit, two weeks after the first visit, and the same treatment was applied. The patient reported later that week that he achieved an orgasm with ejaculation on three separate occasions, each time via self-stimulation. The patient returned for a third visit two weeks later and reported continued success with orgasms and ejaculations. A repeat Oswestry Disability Index was scored at 0% (no disability). The patient was followed up with three months after his initial visit and noted that these improvements were maintained. During the span of treatment and follow-up the patient also did not have any episodes of low back pain.

The patient tolerated all chiropractic treatments well and there were no adverse events in relation to care. The patient provided written consent for the publication of this case and associated images.

## Discussion

This case presents an older man with chronic anorgasmia and anejaculation which improved following manipulation of the lumbar spine provided during three visits over the span of one month and was maintained during an additional two months of follow-up. We suggest that the patient’s sexual dysfunction was caused by neurological dysfunction related to lumbar disc displacement and stenosis, and that his improvement was likewise related to improvement of these conditions.

While the exact pathophysiology of the patient’s anorgasmia and anejaculation was not confirmed with certainty, it is apparent in the literature that disc displacement and stenosis at L5/S1 may result in sexual dysfunction, including anorgasmia and anejaculation, even in the absence of prominent pain [1-4]. While it is not exactly clear when the current patient’s L5/S1 disc displacement and stenosis developed, we suggest these conditions may have become further advanced when he had pneumonia, due to repeated coughing, or prolonged periods of recumbency while ill. In retrospect, walking difficulty, poor balance, and radiculopathy identified via EMG after his illness are consistent with the development of symptomatic lumbar stenosis.

Etiologies outside of the lumbar spine seem to fail to explain the patient’s sexual dysfunction. The patient’s brain, cervical, thoracic spine and pelvic imaging were relatively unremarkable. Pudendal nerve injury was unlikely as the patient had no perineal pain or dysesthesia. A psychological etiology was disputed by the patient’s psychologist and was further questioned when the patient improved after seeing the chiropractor and had already discontinued his selective serotonin reuptake inhibitor. The patient’s sexual dysfunction is also inconsistent with a medication side effect. While selective serotonin reuptake inhibitors (e.g., sertraline) have been reported to cause ejaculatory problems in 10%-30% of individuals, sexual function typically improves by four weeks after discontinuing the medication [8]. Gabapentin has also been implicated with anorgasmia [9]; however, the patient did not change his dose preceding or during chiropractic treatments.

While age-related changes, chiefly a deficiency of testosterone or diminished peripheral nerve function, are potential etiologies of anorgasmia and anejaculation in older men [10-11], these factors did not appear present in the current case. The patient’s testosterone levels were normal, and he reported no loss of penile sensation. In addition, the patient’s symptoms began suddenly, contrasting a gradual decline in function that might be expected with age-related changes [11]. Finally, his anorgasmia and anejaculation improved despite his increasing age.

One potential mechanism for the effects observed in the current case is a spinal manipulation-induced stimulation of the lumbar sympathetic efferent fibers. Preganglionic sympathetic fibers emerge from the rami communicans of the thoracolumbar spine (T10 to L2) and contribute to the sympathetic chain and ganglia that combine to form the hypogastric plexus [2, 5]. Postganglionic fibers then innervate the prostate, bladder neck, vas deferens, seminal vesicles, and vas deferens to mediate sympathetic control of the emission phase of ejaculation [5]. While research on this topic is limited, a systematic review found low-quality evidence that passive spinal mobilization stimulates lumbar sympathetic outflow [6]. This finding was suggested via changes in indirect physiological measures, such as skin conductance, skin temperature, and heart rate in multiple studies [6]. As lumbar mobilization was performed in the current case, it is possible this stimulated sympathetic outflow to the end organs involved in ejaculation. In contrast, erection, which is under parasympathetic control [3], did not improve after spinal manipulation.

It is not clear if our patient’s lumbar degenerative changes impaired the sympathetic innervation required for sexual function. However, a study comparing individuals with lumbar stenosis (most often at L4/5) to an age-matched healthy population found that stenosis was associated with reduction in sympathetic nerve function in the lower extremity, as measured via microneurography [12]. The authors suggested that cauda equina compression caused deficits in sympathetic function [12]. In addition, a cadaveric study reported that anterior lumbar osteophytes of at least 1 cm (as seen in the current case) altered the position of the lumbar sympathetic trunk [13]. However, it was difficult to visualize the sympathetic trunk in the imaging studies in the current case. Further, it is not known if displacement of the sympathetic trunk, by itself, would cause impairments in the sympathetic nerve function. Alternatively, we suggest that inflammatory changes related to disc degeneration could adversely impact the overlying sympathetic chain. In a study using an animal model, nucleus pulposus (i.e., intervertebral disc material) experimentally placed on the anterior aspect of the spine triggered an inflammatory response of the neighboring sympathetic trunk [14]. Based on these findings, the authors hypothesized that anterior displacement of a lumbar intervertebral disc could cause symptoms related to the sympathetic nerves [14].

One potential mechanism for the resolution of anorgasmia and anejaculation in the current case is improvement of sacral radiculopathy. The patient had several neurologic deficits that localized to the sacral nerve roots, for example Achilles hyporeflexia (S1 and S2) and intrinsic toe muscle weakness (S1 to S3). While the patient’s previous EMG/MRI findings were more consistent with L5 radiculopathy, the lower sacral segments S2-4 were not assessed by this testing. These sacral somatic nerves from S2-4 are required for the expulsion phase of ejaculation (i.e., involving external urethral sphincter relaxation, then contraction of the prostate, bulbocavernosus, ishiocavernosus, levator ani, and transverse perineal muscles) [2, 5]. As lumbar stenosis at L5/S1 can compress the descending sacral nerves, it is therefore possible that the present patient had a lesion of these nerves leading to sexual dysfunction.

There are several limitations in the current case. First, we did not have a baseline patient-reported outcome questionnaire which was oriented towards sexual function, such as the International Index of Erectile Function. While the patient had previous EMG, it only examined the lower extremities and paraspinal muscles rather than the perineal/S2-4 distribution muscles. It is unclear if the patient’s erectile dysfunction was related to his lumbar spine or if it was caused by aging or cardiovascular disease, as this component of his sexual dysfunction did not improve. It is unclear if improvements in anorgasmia and anejaculation were related to alleviation of radiculopathy, stimulation of the sympathetic nervous system, or another mechanism, as electrodiagnostic and physiologic testing was not conducted immediately before and after spinal manipulation.

## Conclusions

The current case highlights the improvement of anorgasmia and anejaculation in an older man with lumbar disc displacement and stenosis at L5/S1 after receiving lumbar spinal manipulation. The literature suggests that sexual dysfunction may be relatively common among individuals with lumbar disc displacement and stenosis. Further research is needed to examine the effectiveness of spinal manipulation in alleviating male sexual dysfunction related to lumbar spine disorders. Any such studies should account for confounding variables such as older age and medications.
